# Enhancing methane production using anaerobic co-digestion of waste activated sludge with combined fruit waste and cheese whey

**DOI:** 10.1186/s12896-019-0513-y

**Published:** 2019-03-28

**Authors:** Seyed Mostafa Hallaji, Mohammad Kuroshkarim, Seyede Parvin Moussavi

**Affiliations:** 1 School of Environment, College of Engineering, University of Tehran, Tehran, Iran; 2 Environmental Health Research Center, International Branch of Shahid Sadoughi University of Medical Sciences and Health Services, Yazd, Iran

**Keywords:** Anaerobic co-digestion, Waste activated sludge, Fruit waste, Cheese whey, Methane production, enzymatic activity

## Abstract

**Background:**

Recently, it has been indicated that anaerobic co-digestion of waste activated sludge with other waste streams at wastewater treatment plants is a promising strategy for enhancing methane production and materials recovery. The enhanced methane production can be used as a renewable source of energy in wastewater treatment plants. It can also reduce the amount of greenhouse gas emission in landfilling of the waste streams.

**Results:**

According to the results obtained in this study, anaerobic co-digestion of waste activated sludge with mixed fruit waste and cheese whey improves methane production and the quality of digested sludge in comparison to the anaerobic digestion of waste activated sludge individually. It was indicated that carbon/nitrogen ratio (C/N) in the mixture of waste activated sludge, fruit waste and cheese whey improved considerably, leading to better anaerobic organisms’ activity during digestion. With assessing the activity of protease and cellulase, as the main enzymes hydrolyzing organic matter in anaerobic digestion, it was indicated that co-digestion of waste activated sludge with mixed fruit waste and cheese whey enhances the activity of these enzymes by 22 and 9% respectively. At the end of digestion, the amount of cumulative methane production significantly increased by 31% in the reactor with 85% waste activated sludge and 15% mixed fruit waste and cheese whey, compared to the reactor with 100% waste activated sludge. In addition, chemical oxygen demand (COD) and volatile solid (VS) in digested sludge was improved respectively by 9 and 7% when mixed fruit waste and cheese whey was used.

**Conclusions:**

This study revealed that mixed fruit waste and cheese whey is potentially applicable to anaerobic digestion of waste activated sludge, as fruit waste and cheese whey have high C/N ratio that enhance low C/N in waste activated sludge and provide a better diet for anaerobic organisms. This is of significant importance because not only could higher amount of renewable energy be generated from the enhanced methane production in wastewater treatment plants, but also capital costs of the companies whose waste streams are being transported to wastewater treatments plants could be reduced considerably.

**Electronic supplementary material:**

The online version of this article (10.1186/s12896-019-0513-y) contains supplementary material, which is available to authorized users.

## Background

Nowadays, the importance of wastewater treatment plants in protecting water streams from pollution is widely recognized. Activated sludge process is a conventional method being widely used in wastewater treatment plants throughout the world due to its high efficiency in removing organic matter and simplicity of its application [1]. However, in this process, a large amount of waste activated sludge is produced, which increases sludge treatment costs in wastewater treatment plants. This is important because sludge treatment unit is the most cost-intensive section in wastewater treatment plants, accounting for around 60% of total operation costs [2, 3].Therefore, recent studies have been focused on enhancing digestion of sewage sludge, whereby sludge treatment costs would be reduced [4–9].

Anaerobic digestion is considered as a promising method for stabilizing sewage waste activated sludge, which not only dispenses with aeration facilities and its related capital and operation costs, but it also produces methane as a renewable energy resource in wastewater treatment plants [10–13]. In addition, anaerobic digestion harnesses methane that results in lower greenhouse gas emission when digested sludge is used in farms, forests, and landfills. This is of great importance because around 40% of total greenhouse gas emission in wastewater treatment plants is produced from sludge treatment sections [14–17]. However, an issue attributing to anaerobic digestion of waste activated sludge is its poor biochemical methane potential and slow fermentation process [11, 18–20].

Anaerobic co-digestion of sewage sludge with other waste streams is an environmentally friendly and economically attractive method to overcome low biochemical methane potential of sewage sludge [13, 21–28]. Hublin et al. [21] revealed that anaerobic co-digestion of cheese whey and cow manure reduces CO_2_ and CH_4_ emissions by 3.5 ktonnes CO_2_/year and 5.7 ktonnes CO_2_-eq/year respectively. They, also, indicated that a medium-scale biogas plant is profitable for 12 and 15 year effectuation periods. Sewage waste activated sludge has a C/N ratio of around 10, but the suitable ratio for anaerobic digestion of organic matter is around 20 to 30 [29]. Therefore, the low C/N in waste activated sludge results in imbalanced diet for anaerobic organisms and low biodegradability of organic matter [30]. C/N ratio in waste activated sludge is low because it mainly consists of microbial cells leading to high nitrogen content [31]. High organic loading rate (OLR) can also cause low C/N ratio due to ammonia accumulation that produces inhibitory effects on anaerobic organisms’ activity [32, 33]. Consequently, anaerobic co-digestion of waste activated sludge with other waste streams, which have higher carbon content, can provide a balanced diet for anaerobic organisms’ activity. Hosseini Koupaie et al. [34] revealed that anaerobic co-digestion of fruit-juice waste and municipal sludge reduces total net costs up to 37%. Anh et al. [35] investigated the effect of anaerobic digestion of sewage sludge with carbon-rich organic waste on microbial community. They showed that adding higher proportion of carbon-rich organic waste reduces diversity of microbial community, but increases biogas production. Fitamo et al. [36] demonstrated that co-digestion of food waste, grass clippings, garden waste with mixed municipal sludge enhances methane production by 35–48% and decreases hydraulic retention time (HRT) to 15 days. Maragkaki et al. [23] revealed that anaerobic co-digestion of sewage primary sludge (95%) with mixed olive mill and cheese whey wastewater (5%) in continuous reactors can enhance methane yield and biogas production around 127% and improve organic removal accordingly.

In this study, we hypothesized that combined cheese whey and fruit waste could enhance C/N ratio in waste activated sludge significantly, leading to better anaerobic microorganisms’ performance, meanwhile provide simultaneous digestion of these waste streams, which reduces related costs and environmental impacts in comparison to individual digestion of the waste streams. This is the first investigation focusing on anaerobic co-digestion of mixed cheese whey and fruit waste with sewage waste activated sludge. Substrate characterizations in different proportions of waste activated sludge with mixed fruit waste and cheese whey were measured before conducting the anaerobic co-digestion process. During anaerobic co-digestion, key hydrolytic enzymes activity, biogas and methane production, volatile solid (VS) and chemical oxygen demand (COD) were measured. In this study, different ratios of waste activated sludge and mixed fruit waste and cheese whey were used in biochemical methane potential tests, so as to investigate the feasibility of enhancing methane production and quality of the digested waste streams in the anaerobic co-digestion.

## Results

### Substrates and inoculum

In this study waste activated sludge and inoculum were collected from the south wastewater treatment of Tehran, the biggest wastewater treatment plant of the Middle East. This plant treats wastewater of 4,200,000 people living in central and southern parts of Tehran, Iran. In this study, the waste activated sludge was collected from belt thickener and the inoculum was collected from six anaerobic digesters being operated in mesophilic condition at this plant. The different characterizations of the collected sludge such as total chemical oxygen demand (TCOD), soluble chemical oxygen demand (SCOD), volatile solid (VS), volatile suspended solid (VSS), total solid (TS), total suspended solid (TSS), alkalinity, pH and C/N were measured as soon as the sludge was conveyed to the university’s laboratory (Table 1).

**Table 1:** Characterizations of the substrates and inoculum

Characterizations	Waste activated sludge	Fruit waste	Cheese whey	inoculum
TCOD (g/l)	52.10 ± 0.70	32.51 ± 0.71	92.45 ± 0.82	38.21 ± 0.02
SCOD (g/l)	4.02 ± 0.50	12.45 ± 0.30	53.65 ± 0.91	3.550 ± 0.078
VS (g/l)	33.12 ± 0.52	20.52 ± 0.52	70.12 ± 0.81	25.15 ± 0.031
VSS (g/l)	30.01 ± 0.36	11.64 ± 0.12	32.10 ± 0.40	23.11 ± 0.09
TS (g/l)	41.25 ± 0.62	23.12 ± 0.48	84.34 ± 0.79	32.34 ± 0.012
TSS (g/l)	36.8 ± 0.56	14.43 ± 0.21	39.66 ± 0.57	28.45 ± 0.12
pH	6.54 ± 0	5.71 ± 0	5.88 ± 0	7.72 ± 0
C/N	10 ± 0.2	19 ± 0.4	35 ± 0.5	7 ± 0.3
Alkalinity (mg CaCO3/l)	3050 ± 25	1100 ± 25	2550 ± 44	3710 ± 52

Cheese whey used in this study was collected from a local cheese-producing factory, working with traditional cheese producing technologies in the same region. Fruit waste was also collected from fruit stores near the university’s laboratory. After shredding and homogenizing, the fruit waste was diluted with distilled water to reduce its dry solid (DS) to around 2 g/L, so as to set overall DS of the mixtures (substrate and inoculum) at 3 to 9%, which is suggested by Metcalf and Tchobanoglou [37] as the best DS in anaerobic digesters for uniform nutrient and heat distribution, and proper mixers’ performance. The fruit waste consisted of homogenized apple, tomato, carrot, orange and potato with equal proportion (based on weight). Characterizations of the cheese whey and fruit waste were measured as soon as they was conveyed to the university’s laboratory (Table 1).

For assessing the influence of adding mixed fruit waste and cheese whey to anaerobic digestion of waste activated sludge, four ratios of waste activated sludge with mixed cheese whey and fruit waste were considered; A mixture with 100% waste activated sludge was considered as a control, the three other ratios were 95, 90 and 85% waste activated sludge with 5, 10 and 15% mixed fruit waste and cheese whey respectively. Characterizations of the mixtures with different ratios were measured as soon as the waste streams were completely mixed with each other (Table 2).

**Table 2:** Characterizations of mixed substrates

Waste activated sludge: mixed fruit and cheese whey	100:0	95:5	90:10	85:15
COD (g/l)	52.1 ± 0.06	53.12 ± 0.06	54.78 ± 0.06	55.31 ± 0.06
SCOD (g/l)	4.020 ± 0.08	6.23 ± 0.11	7.12 ± 0.09	8.51 ± 0.21
VS (g/l)	33.12 ± 0.02	34.41 ± 0.82	34.91 ± 0.92	35.33 ± 0.82
VSS (g/l)	30.01 ± 0.06	28.91 ± 0.52	29.45 ± 0.61	30.27 ± 0.4
TS (g/l)	41.25 ± 0.02	42.68 ± 0.55	44.11 ± 0.71	44.72 ± 0.52
TSS (g/l)	36.8 ± 0.06	35.96 ± 0.72	35.21 ± 0.81	34.87 ± 0.83
pH	6.54 ± 0	6.51 ± 0	6.49 ± 0	6.46 ± 0
C/N	10 ± 0.2	13.5 ± 0.3	15.12 ± 0.25	16.33 ± 0.52
Alkalinity (mg CaCO3/l)	3050 ± 25	2651 ± 0.43	2570 ± 56	2450 ± 63

### Key hydrolytic enzymes activity

Protease and cellulase play an important role in decomposition of organic matter and provision of more readily biodegradable organic matter for anaerobic organisms. Cellulase catalyzes hydrolyze of polysaccharides to monoses and protease degrades proteins to amino acids [12]. In this study, the activity of protease and cellulase, as two key hydrolytic enzymes, were measured in the biochemical methane potential reactors with and without mixed fruit waste and cheese whey (Table 3). According to the data obtained, both protease and cellulase activities increased significantly (*p* < 0.05) in the bioreactors containing mixed cheese whey and fruit waste in comparison to the control bioreactors. The highest increase in the activity of protease and cellulase was observed in the reactor containing 85% waste activated sludge and 15% mixed fruit and cheese whey that accounted respectively for 22 and 10% in comparison to the control reactor. Followed by that, the reactor containing 90% waste activated sludge and 10% cheese whey had the second highest protease and cellulase activity with 17 and 6% higher than the control.

**Table 3:** Protease and cellulase activity in different proportions of mixed cheese whey and fruit waste

Waste activated sludge: mixed fruit and cheese whey (%)	100/0	95/5	90/10	85/15
Protease (mm)	21.0 ± 0.3	23.5 ±0.7	24.6 ± 0.6	25.6 ± 0.8
Cellulase (mm)	26.5 ± 0.4	27.5 ± 0.6	28.1 ± 0.4	29.3 ± 0.8

### Daily biogas and methane production

**Fig. 1:**
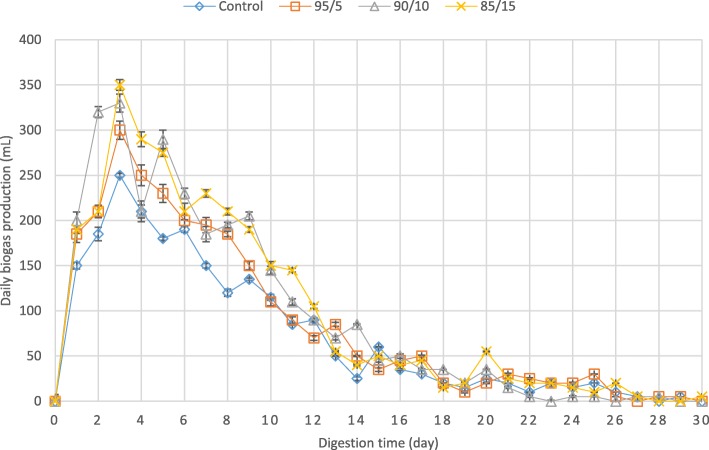
Daily biogas production from the bioreactors during digestion time. Error bars represent standard errors obtained from triplicate measurements

Daily biogas production from different bioreactors was measured during anaerobic digestion. The amount of daily biogas production enhanced in all of the bioreactors containing mixed fruit waste and cheese whey in comparison to the control bioreactor (Fig. 1). The maximum daily biogas was achieved in the bioreactor containing 85% waste activated sludge and 15% mixed cheese whey and fruit waste with 350 mL in the third day of digestion (Additional file 1). Analogously, cumulative biogas production, which can be obtained by calculating the area below each chart, was the highest in this bioreactor with 2970 mL at the end of digestion process (30th day). This was 33% higher than the cumulative biogas produced from the control bioreactor with 2225 mL at the end of digestion process.

**Fig. 2:**
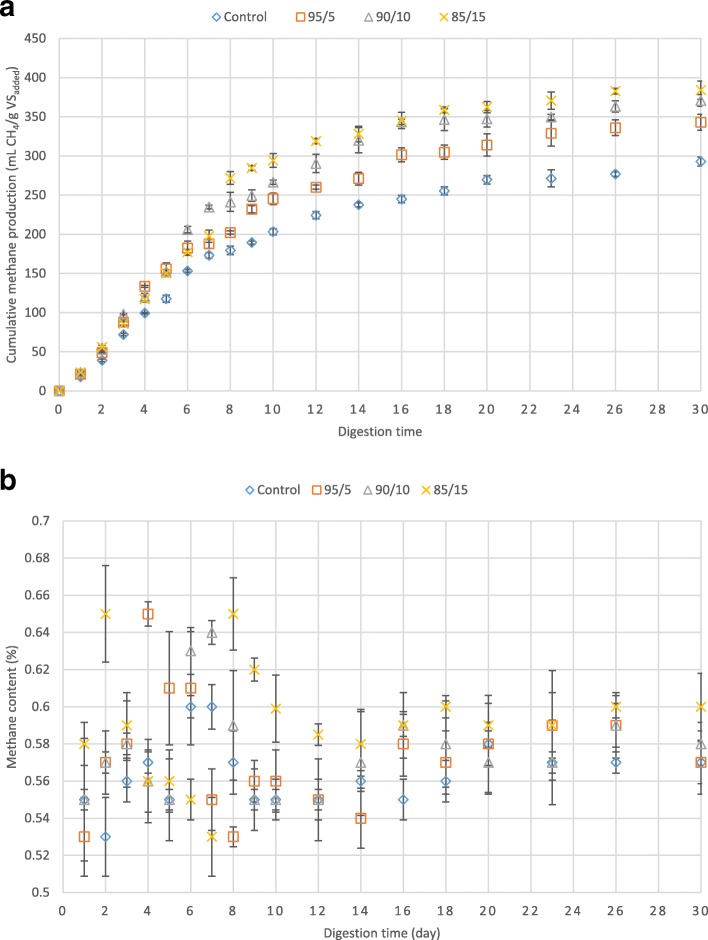
**a** Cumulative methane production from the bioreactors during digestion time. Error bars represent standard error obtained from triplicate tests. **b** Methane content during anaerobic digestion. Error bars represent standard error obtained from triplicate tests

Methane production from the bioreactors was regularly measured during the anaerobic digestion. According to the data obtained, the amount of methane production from the bioreactors containing cheese whey and fruit waste enhanced considerably in comparison to the control bioreactor (Fig. 2-a). For normalizing the data, the produced methane (mL) was divided by the added volatile solids (gram) to each bioreactors. The highest amount of methane production was achieved in the bioreactor containing 15% mixed fruit waste and cheese whey with 85% waste activated sludge that accounted for 384.1 mL CH_4_/g VS _added_ (Additional file 1). This is 31% higher than that of the control bioreactor with 292.76 mL CH_4_/g VS _added_ and 5% higher than that of the bioreactor containing 90% waste activated sludge and 10% mixed cheese whey and fruit waste (with 370.77 CH_4_/ g VS _added_)_._

The amount of methane content during the anaerobic digestion fluctuated between 53 and 65% (Fig. 2-b). Assessing the mean value of methane content from different bioreactors, it was indicated that the mean value in the bioreactors containing mixed cheese whey and fruit waste during digestion period increased by 3%, reaching to 59% from 56%.

### Removal of organic matter

**Fig. 3:**
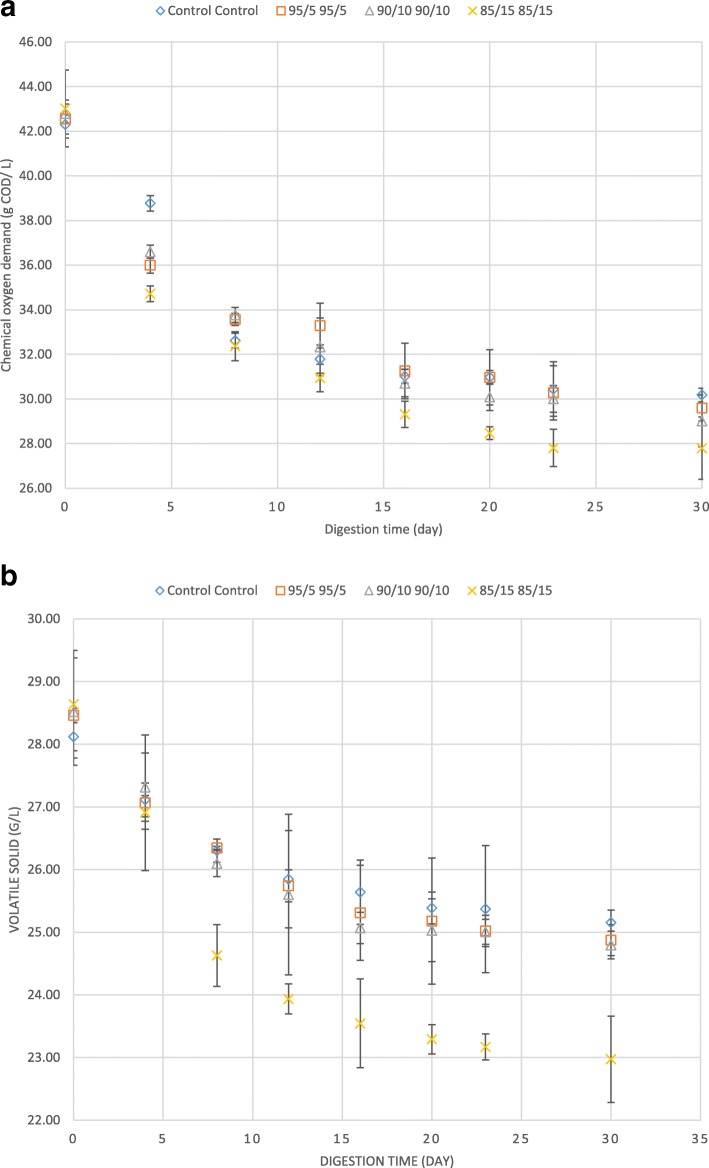
**a** Chemical oxygen demand from the bioreactors during digestion time. Error bars represent standard error obtained from triplicate tests. **b** Volatile solid from the bioreactors during digestion time. Error bars represent standard error obtained from triplicate tests

The amount of COD and VS in digested sludge play an important role in applicability of the sludge to agricultural lands and forests as they should meet the local environmental criteria. In addition, VS is an important characteristic affecting transportation costs of sludge [38] and it represents the metabolic status of microbial community in anaerobic system [39]. Therefore, it is imperative to reduce the costs of sludge management in wastewater treatment plants that accounts for around 60% of total operation costs of wastewater treatment plants [2, 20]. In this study, the amount of VS and COD were regularly measured during the anaerobic digestion process. It was demonstrated that the removal efficiency of COD and VS enhanced in the bioreactors containing mixed cheese whey and fruit waste (Fig. 3-a and -b respectively). The highest improvement achieved in the bioreactor containing 15% mixed cheese whey and fruit waste with 85% waste activated sludge, in which COD and VS removal enhanced by 9 and 7% in comparison to the control.

## Discussion

The enhancement of enzymes’ activity in the reactors contained cheese whey and fruit waste could be mainly attributed to the balanced C/N ratio, which provides higher amount of nutrients for microbial activity. Analogously, Feng et al. [30] revealed that the activity of protease, as a protein degrading enzyme, increases by 25% when waste activated sludge (with C/*N* = 7.1) is anaerobically co-digested with rice (C/*N* = 20), corroborating the effectiveness of balanced C/N in improving the activity of key hydrolytic enzymes.

The enhancement of biogas was marked from the first day of digestion process, indicating the positive effect of hydrolytic key enzymes activity in providing more readily biodegradable organic matter for better microbial activity. Higher soluble chemical oxygen demand in these reactors in comparison to the control provided higher amount of readily biodegradable organic matter for anaerobic organisms that may lead to increase of biogas production. However, in previous studies it was indicated that extremely high SCOD may lead to accumulation of volatile fatty acids, making a delay in biogas production or reducing pH of bioreactors [4, 40, 41].

The methane production enhanced as the proportion of mixed cheese whey and fruit waste increased. This is probably attributed to more balanced nutrients available to microbial community, positive synergism effect, and higher hydrolytic enzymes activity (Table 3). The enhancement in the bioreactors containing mixed cheese whey and fruit waste was significant (*p* < 0.05) in comparison to the control, corroborating the effectiveness of anaerobic co-digestion of waste activated sludge with mixed cheese whey and fruit waste in enhancing methane production. The enhanced methane is of paramount importance because not only does it enhance renewable energy generation in wastewater treatment plants, but it also reduces methane emission to the atmosphere, as a major greenhouse gas emission [17]. However, the population means of methane content was not significantly different in the bioreactors containing 5 and 10% mixed cheese whey and fruit waste (*p* = 0.41 > 0.05), but that of the bioreactor contained 15% cheese whey and fruit waste was of significant difference (*p* = 0.01 < 0.05). This confirms that mixed cheese whey and fruit waste in higher proportion can be effective in improving methane content of the biogas. In comparison to this study, higher methane content (67%) was achieved in Maragkaki et al. [23] study, in which sewage sludge was co-digested with mixed cheese whey, food waste and olive mill wastewater. Also, Habiba et al. [42] assessed anaerobic co-digestion of waste activated sludge with fruit and vegetable waste, in which methane content fluctuated around 60% in all of the bioreactors. The difference in results can be attributed to wastes specifications and operation conditions employed in these studies, compared to the current study. In this study, methane production from the bioreactors containing mixed cheese whey and fruit waste enhanced considerably from the first day of digestion, indicating lower lag time in these bioreactors in comparison to the control. Analogous trend was observed in Zou et al. [22] study, in which lower lag time (0.2 day) was achieved in anaerobic co-digestion of residual sludge and lignocellulosic wastes. Also, Callaghan et al. [43] revealed that cattle slurry with fruit waste can enhance methane production to 450 mL CH_4_/g VS _added_ and adding chicken manure to this mixture declines methane production and VS reduction due to the accumulation of free ammonia.

The improved removal of organic matter can be linked to higher biogas and methane production, which lead to higher degradation of organic matter during anaerobic co-digestion of waste streams. The improved VS and COD are of great significance because not only does it reduce the transportation costs of digested waste streams, but it also paves the way for having integrated, sustainable system for reusing digested wastes in agricultural lands and forests. Compared to the current research, the amount of VS reduction was higher in Habiba et al. [42] study, in which waste activated sludge with fruit and vegetable waste was anaerobically digested in sequencing batch reactors.

In spite of the significant results this study represents, applying new methods to sludge treatment section of wastewater treatment plants entails inspecting long-term effect of the anaerobic co-digestion on microbial community and assessing economic viability of the proposed methods. Therefore, in prospective research, the effect of the mixed waste streams on microbial behaviour, economic viability, and environmental impacts of the proposed method should be put into perspective.

## Conclusions

This study investigated the viability of enhancing anaerobic digestion of waste activated sludge using mixed fruit waste and cheese whey. It was revealed that anaerobic co-digestion of waste activated sludge with mixed fruit and cheese whey could stimulate hydrolytic enzymes activity and providing better nutrients balance for microbial communities, whereby not only methane production increases, but also organic matter removal enhances at the end of digestion process. The increased methane production is of paramount importance because higher amount of renewable energy could be produced from anaerobic digestion. It also reduces methane emission from sludge treatment units with harnessing methane, as a major greenhouse gas, and converting it to renewable energy. The enhanced removal of organic matter paves the way for having more integrated system via heightening quality of the digested waste streams, which increases applicability of the digested wastes to agricultural lands and reduces transportation costs of the wastes in wastewater treatment plants.

## Methods

### Analytical methods

TS, VS, TSS, VSS, COD, SCOD and alkalinity were measured according to standard methods for the examination of water and wastewater [44]. To separate solid particles from liquid phase, samples were centrifuged at 10000 rpm for 30 min, then the supernatant was filtered through 0.45 μm pore size glass fiber filters, using Buchner funnel and vacuum pump. C/N ratio was measured in a commercial laboratory. Total nitrogen (TN) was measured according to persulfate digestion method using Hach spectrophotometer. Total carbon (TC) was measured by catalytic oxidation on a total organic carbon (TOC) Euroglace analyser.

Protease and cellulase activities were measured according to well agar diffusion method [45, 46], in which carboxymethyl cellulase agar (CMCA) and plate count skim milk agar (PSMA) were used as growth medium for protease and cellulose enzymes. Methanogens affect protease and cellulase activity by consuming hydrolyzed organic matter, so for removing the errors caused by methanogens in measuring enzymatic activity, sludge samples were heated at 102 °C for 30 min prior to well agar diffusion tests and the methanogens were removed from the samples [47, 48]. After removing methanogens, the reactors were flushed with N_2_ gas. Then, they were sealed and put into water bath (36 ± 1 °C) for 72 h. Then the samples were used in well agar diffusion method.

Biogas and methane production were measured regularly during the digestion process. For measuring biogas production, liquid displacement method was employed, in which liquid barrier was 100% saturated with NaCl and acidified with H_2_SO_4_ (pH = 2), so as to reduce dissolution of CO_2_ and CH_4_ into the liquid barrier [49]. Methane production was measured with gas chromatography (GC), using thermal conductivity detector (TCD), and helium as carrier gas. The biogas was collected in Tedlar gas bags prior to use in GC tests.

### Biochemical methane potential tests

**Fig. 4:**
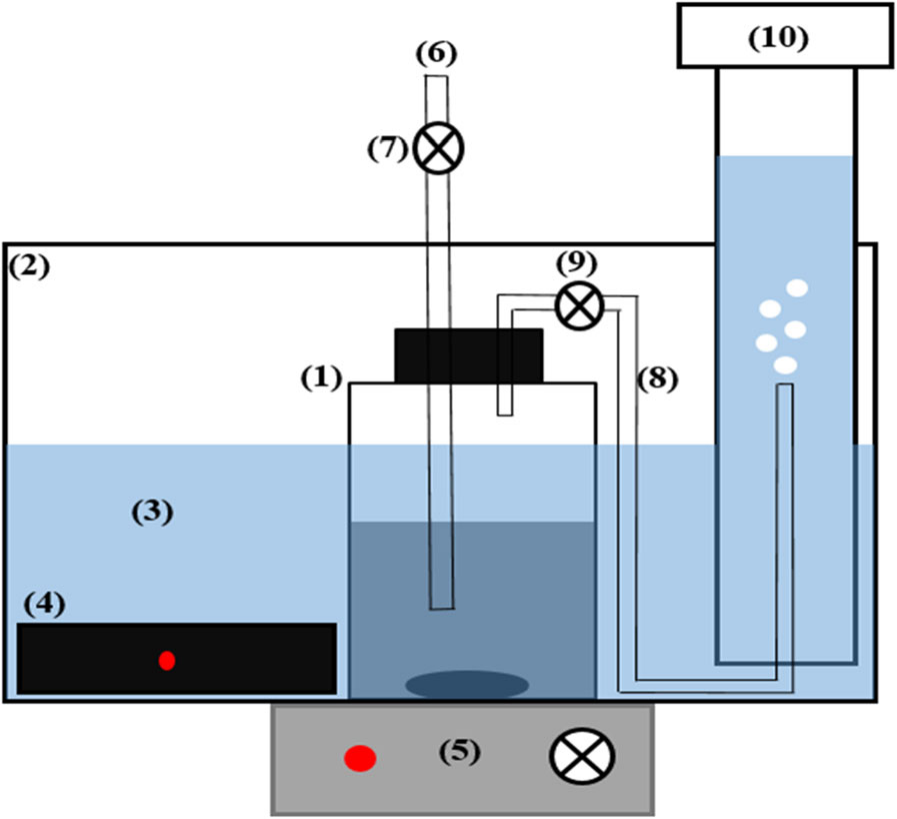
Schematic of experimental system. 1) BMP reactor, 2) aquarium 3) saturated and acidified water, 4) automatic heater, 5) magnetic stirrer, 6) sampling pipe, 7) control valve, 8) biogas collecting pipe, 9) biogas control valve, 10) graduated cylinder

Biochemical methane potential tests were carried out in 1000 mL bioreactors with working volume of 500 mL (Fig. 4). Fruit waste and cheese whey were mixed (50%:50% *V*/V) prior to mix with waste activated sludge. Then, the mixed fruit waste and cheese whey was combined with waste activated sludge as substrate for the inoculums. The ratio between the inoculum and substrate (I/S) was considered as 2 in this study for better activity of anaerobic organisms [50]. A control reactor without mixed fruit waste and cheese whey was considered for comparing different bioreactors. The pH of substrates was set at 7 and their temperature was increased to 37 °C prior to mix with the inoculums, so as to prevent pH and temperature shock to anaerobic organisms. After the substrates and inoculums were mixed, they were flushed with N_2_ gas for 2 min (1 L/min) and precisely sealed for creating strict anaerobic condition. The bioreactors were kept in warm water bath, heated by automatic heaters at 36 ± 1 °C during the anaerobic digestion. For adequate distribution of nutrients to various microbial communities and making uniform temperature in different parts of each bioreactor, they were constantly blended with magnetic stirrers at 100 rpm. The measurements were performed for 30 days, when no biogas production was detected from the bioreactors.

### Data analyses

The significance of difference between the studied parameters was specified with one-way factor Analysis of Variance ANOVA and the significance level was considered as *p* < 0.05. Data analyses and graph processing were conducted with Microsoft Excel software (2010).

## Additional file

Additional file 1: **Table S1.** Daily biogas production during digestion process (average of triplicate tests). **Table S2.** Cumulative methane production during digestion process (average of triplicate tests). **Figure S1.** Gas chromatography samples. (DOCX 50 kb)

## Abbreviations

CMCA: Carboxymethyl cellulase agar
COD: Chemical oxygen demand
GC: Gas chromatography
PSMA: Plate count skim milk agar
SCOD: Soluble chemical oxygen demand
TC: Total carbon
TCD: Thermal conductivity detector
TN: Total nitrogen
TOC: Total organic carbon
TS: Total solid
TSS: Total suspended solid
VS: Volatile solid
VSS: Volatile suspended solid
