# Supplementary material for: Enhancing methane production using anaerobic co-digestion of waste activated sludge with combined fruit waste and cheese whey
Source: BMC Biotechnol. 2019 Mar 28;19:19. doi: 10.1186/s12896-019-0513-y (PMC6437933; doi:10.1186/s12896-019-0513-y)

**Additional Files**

Table 1-S. Daily biogas production during digestion process (average of triplicate tests)

| Day | Control | 95/5 | 90/10 | 85/15 |
| --- | --- | --- | --- | --- |
| 0 | 0 | 0 | 0 | 0 |
| 1 | 150 | 185 | 200 | 190 |
| 2 | 185 | 210 | 320 | 210 |
| 3 | 250 | 300 | 330 | 350 |
| 4 | 210 | 250 | 210 | 290 |
| 5 | 180 | 230 | 290 | 275 |
| 6 | 190 | 200 | 230 | 210 |
| 7 | 150 | 195 | 185 | 230 |
| 8 | 120 | 185 | 195 | 210 |
| 9 | 135 | 150 | 205 | 190 |
| 10 | 115 | 110 | 145 | 150 |
| 11 | 85 | 90 | 110 | 145 |
| 12 | 90 | 70 | 90 | 105 |
| 13 | 50 | 85 | 70 | 55 |
| 14 | 25 | 50 | 85 | 40 |
| 15 | 60 | 35 | 45 | 50 |
| 16 | 35 | 45 | 50 | 40 |
| 17 | 30 | 50 | 35 | 45 |
| 18 | 20 | 20 | 35 | 15 |
| 19 | 15 | 10 | 20 | 20 |
| 20 | 25 | 20 | 35 | 55 |
| 21 | 20 | 30 | 15 | 25 |
| 22 | 10 | 25 | 5 | 20 |
| 23 | 20 | 20 | 0 | 20 |
| 24 | 15 | 20 | 5 | 15 |
| 25 | 20 | 30 | 5 | 10 |
| 26 | 10 | 5 | 0 | 20 |
| 27 | 5 | 0 | 5 | 5 |
| 28 | 0 | 5 | 5 | 0 |
| 29 | 5 | 5 | 0 | 0 |
| 30 | 0 | 0 | 0 | 5 |

Table 2-S. Cumulative methane production during digestion process (average of triplicate tests)

| Day | Control | 95/5 | 90/10 | 85/15 |
| --- | --- | --- | --- | --- |
| 0 | 0 | 0 | 0 | 0 |
| 1 | 18.1 | 21.3 | 23.8 | 23.8 |
| 2 | 38.9 | 48.9 | 53.1 | 56.0 |
| 3 | 71.8 | 87.6 | 97.9 | 85.8 |
| 4 | 99.4 | 133.5 | 120.0 | 117.1 |
| 5 | 117.6 | 155.8 | 152.4 | 150.3 |
| 6 | 153.3 | 182.3 | 205.9 | 177.2 |
| 7 | 173.0 | 187.7 | 234.8 | 197.6 |
| 8 | 179.4 | 202.2 | 241.4 | 271.8 |
| 9 | 189.4 | 231.9 | 249.4 | 284.6 |
| 10 | 203.2 | 245.3 | 266.7 | 294.3 |
| 12 | 224.3 | 260.1 | 290.5 | 319.0 |
| 14 | 237.6 | 271.2 | 320.2 | 328.1 |
| 16 | 244.8 | 301.3 | 343.5 | 345.2 |
| 18 | 255.4 | 304.8 | 346.5 | 358.8 |
| 20 | 269.6 | 314.0 | 347.3 | 362.4 |
| 23 | 271.3 | 329.0 | 349.8 | 370.7 |
| 26 | 276.9 | 336.0 | 363.3 | 382.8 |
| 30 | 292.8 | 343.0 | 370.8 | 384.1 |

**Fig 1-S.** Gas chromatography samples.


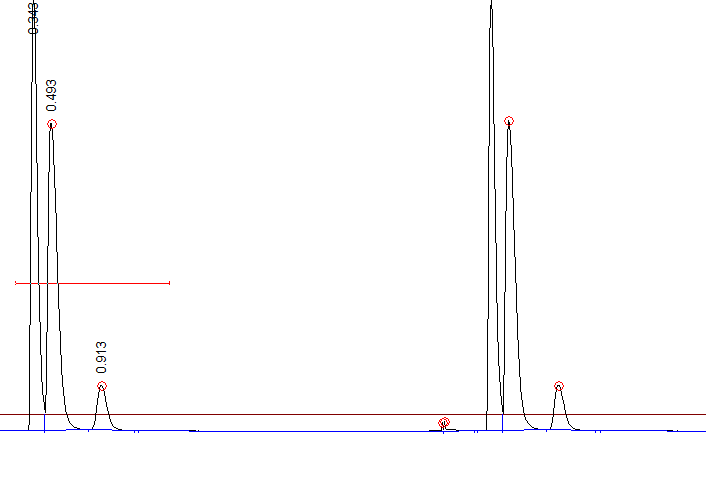


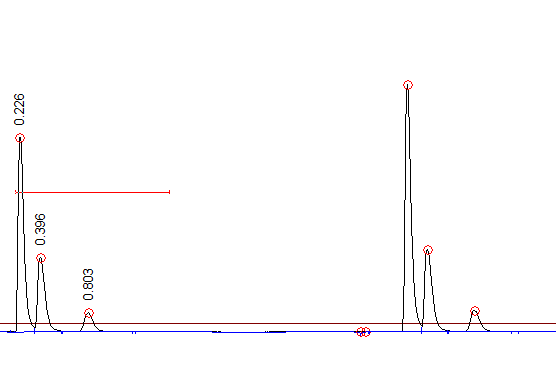


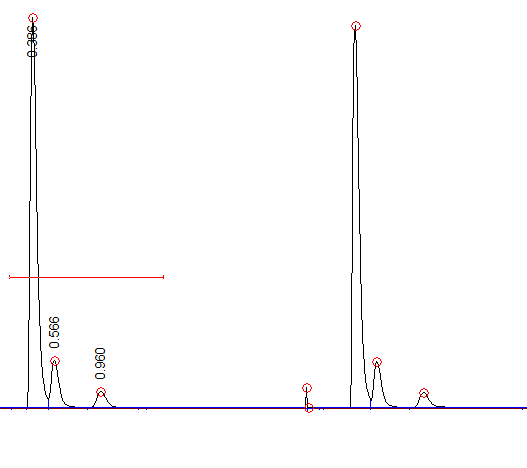

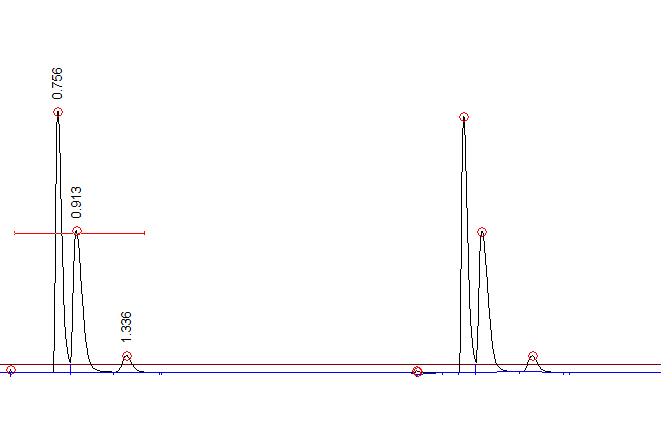


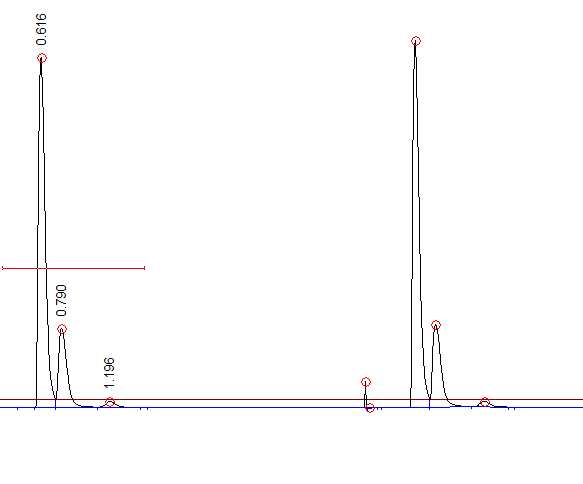

Supplement: Supplementary file 1 — Table S1. Daily biogas production during digestion process (average of triplicate tests). Table S2. Cumulative methane production during digestion process (average of triplicate tests). Figure S1. Gas chromatography samples. (DOCX 50 kb) [file 12896_2019_513_MOESM1_ESM.docx]
